# Human Milk-Derived Levels of let-7g-5p May Serve as a Diagnostic and Prognostic Marker of Low Milk Supply in Breastfeeding Women

**DOI:** 10.3390/nu15030567

**Published:** 2023-01-21

**Authors:** Steven D. Hicks, Desirae Chandran, Alexandra Confair, Anna Ward, Shannon L. Kelleher

**Affiliations:** 1 Department of Pediatrics, Penn State College of Medicine, Hershey, PA 17033, USA; 2 Department of Biomedical and Nutritional Sciences, University of Massachusetts Lowell, Lowell, MA 01854, USA

**Keywords:** lactation, human milk, breastfeeding, miRNAs, low milk supply

## Abstract

Low milk supply (LMS) is associated with early breastfeeding cessation; however, the biological underpinnings in the mammary gland are not understood. MicroRNAs (miRNAs) are small non-coding RNAs that post-transcriptionally downregulate gene expression, and we hypothesized the profile of miRNAs secreted into milk reflects lactation performance. Longitudinal changes in milk miRNAs were measured using RNAseq in women with LMS (*n* = 47) and adequate milk supply (AMS; *n* = 123). Relationships between milk miRNAs, milk supply, breastfeeding outcomes, and infant weight gain were assessed, and interactions between milk miRNAs, maternal diet, smoking status, and BMI were determined. Women with LMS had lower milk volume (*p* = 0.003), were more likely to have ceased breast feeding by 24 wks (*p* = 0.0003) and had infants with a lower mean weight-for-length z-score (*p* = 0.013). Milk production was significantly associated with milk levels of miR-16-5p (R = −0.14, adj *p* = 0.044), miR-22-3p (R = 0.13, adj *p* = 0.044), and let-7g-5p (R = 0.12, adj *p* = 0.046). Early milk levels of let-7g-5p were significantly higher in mothers with LMS (adj *p* = 0.0025), displayed an interaction between lactation stage and milk supply (*p* < 0.001), and were negatively related to fruit intake (*p* = 0.015). Putative targets of let-7g-5p include genes important to hormone signaling, RNA regulation, ion transport, and the extracellular matrix, and down-regulation of two targets (PRLR and IGF2BP1/IMP1) was confirmed in mammary cells overexpressing let-7g-5p in vitro. Our data provide evidence that milk-derived miRNAs reflect lactation performance in women and warrant further investigation to assess their utility for predicting LMS risk and early breastfeeding cessation.

## 1. Introduction

Low milk supply is associated with early breastfeeding cessation. Nearly 40% of breastfeeding women cite concerns over low milk supply as a primary reason for not meeting their breastfeeding goals [1]. The etiology of suboptimal lactation is clearly multifaceted. Abnormal breast conditions and previous breast surgeries [2] are structural factors that contribute to suboptimal lactation. In addition, numerous social, psychological, and behavioral factors are associated with early breastfeeding cessation [3,4,5,6]. Several studies reported associations between maternal metabolic conditions such as malnutrition [7], excessive maternal fat mass [8], and gestational diabetes [9] and milk supply. Moreover, we and others determined that genetic variations in genes critical for mammary gland function (i.e., prolactin (*PRL*) [10], prolactin receptor (*PRLR*) [11], ZnT2 (*SLC30A2*) [12], and milk fat globule-epidermal factor 8 protein (*MFGE8*) [13]) are associated with the ability to produce milk, providing evidence that biological factors are also responsible for low milk supply.

MicroRNAs (miRNAs) are small, non-coding nucleic acid sequences (~22 nucleotides) that post-transcriptionally regulate gene expression by binding to specific mRNA targets and either inhibiting translation or promoting degradation, thus affecting corresponding protein expression. Strong evidence indicates miRNAs control normal physiology and miRNAs have been associated with various pathological states [14]. MiRNAs are found in all bodily fluids, including saliva, plasma, and urine [15,16], although milk is one of the richest sources of miRNAs in humans, containing ~1400 mature miRNAs [17]. Many of these miRNAs are released by secreting mammary epithelial cells (MECs) in exosomes. Mammary gland miRNA profiles differentiate discrete stages of mammary gland development in rodents and dairy animals, and parallel gene expression required for ion transport, G protein signaling, translation, and intracellular protein transport, and oxidative phosphorylation [18,19,20]. Several previous studies suggested the profile of human milk miRNAs may reflect breast function [17,21]. Consistent with this hypothesis, we and others [22,23] previously showed that several maternal factors associated with milk production and composition (i.e., diet, genotype, preterm birth, and stage of lactation) are associated with the profile of milk-derived miRNAs, implicating milk miRNAs as bioreporters of lactation performance in humans [24]. Here we hypothesized that specific milk-derived miRNAs are associated with low milk supply, and using a genome-wide computational approach, identified milk-derived miRNAs that may serve as novel genetic drivers or reporters of low milk supply and confirmed regulation of two targets in MECs in vitro.

## 2. Materials and Methods

### 2.1. Participants

**Figure 1:**
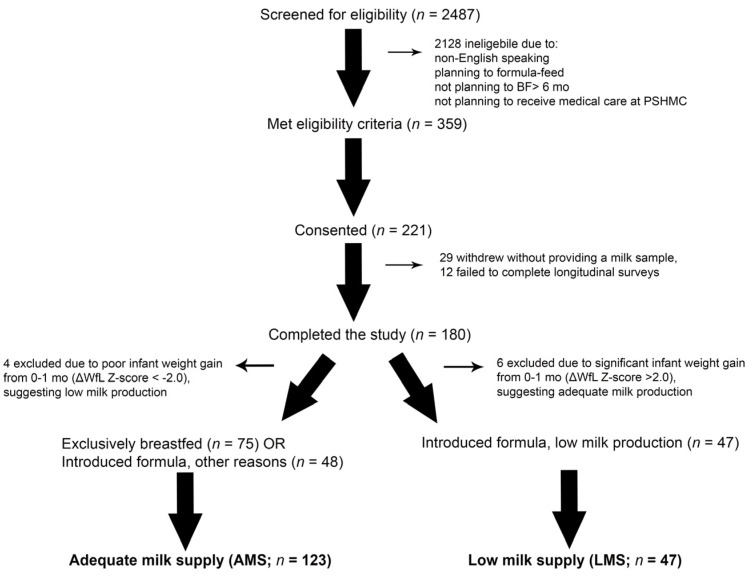
CONSORT Diagram. Research staff screened 2487 mother–infant dyads, approached 359 eligible dyads, and obtained consent from 221 dyads. There were 180 mothers that provided at least one milk sample and completed sufficient longitudinal surveys to determine whether they experienced low milk supply (LMS) or adequate milk supply (AMS). There were 4 or 6 mothers excluded from each group for excessive infant weight gain or weight loss (defined as a change in weight-for-length (WfL) Z-score > 2.0), which suggested milk production may have been over- or under-estimated. This left 47 mothers with LMS, and 123 mothers with AMS. Of the 123 mothers with AMS, 48 introduced formula into their infant’s diet prior to 12 months for reasons other than concerns about milk supply.

This longitudinal cohort study involved a convenience sample of 221 women, ages 19–42 years. Mothers of full-term, singleton infants (37–42 weeks gestation) who planned to breastfeed at least six months were eligible. Exclusion criteria and the study design were previously reported [13]. Participants were dichotomized into two groups: low milk supply (LMS) or adequate milk supply (AMS) as we have previously reported. Ultimately, 47 women with LMS were compared against mothers with AMS (*n* = 123) who either maintained exclusive breast feeding throughout the duration of the study or reported formula introduction for reasons other than “decreased or low breastmilk production” (Figure 1).

### 2.2. Participant Characteristics

Participant characteristics were collected via electronic surveys administered by research staff at enrollment. Survey responses were confirmed through review of the medical record where possible. The following medical and demographic characteristics were collected: maternal age, maternal race, parity, pre-pregnancy body mass index, tobacco use, maternal educational level, marital status, duration of previous breastfeeding, infant gestational age, infant sex, and infant birth weight as previously reported [13]. No women reported the use of galactagogue supplements.

Maternal nutrition was assessed alongside each milk collection (at 1, 4, 16 wks) through electronic administration of the Dietary Screener Questionnaire (DSQ), developed as part of the National Health and Nutrition Examination Survey (NHANES) [25]. Published guidelines were used to compute consumption of fruit, vegetables, dairy, added sugars, and calcium. Infant feeding characteristics were collected through electronic administration of the modified Infant Feeding Practices survey (IFP), and as we have previously reported, milk production was approximated based on maternal report of pumping volumes and infant feeding practices [13]. Mothers who were unable to estimate milk production volumes (*n* = 57/340 data-points; 16%) were excluded from analyses of milk production. For mothers who reported infant weaning (or failed to provide a milk sample due to low milk supply; *n* = 25), a milk production volume of 0 oz/day was assigned. Infant weight and length were abstracted from the medical record at birth and four weeks post-delivery. For each infant, the change in weight-for-length Z-score was calculated at each time point using standardized curves from the World Health Organization.

### 2.3. Milk Collection

One milk sample was collected from each mother at 1, 4, and 16 wks post-delivery (or until breastfeeding ceased). The 170 mothers provided 453 milk samples: 170 samples in the first wk post-delivery, 158 samples 4 wks post-delivery, and 125 samples 16 wks post-delivery. Maternal milk (1–5 mL) was manually expressed from a sterilized nipple surface into RNAse-free tubes prior to feeding (i.e., fore-milk). Foremilk samples were exclusively collected from the same breast to minimize confounding impacts of fore- and hind-milk differences [17]. Samples were immediately transferred to −20 °C, underwent one freeze–thaw cycle for aliquoting, and stored at −80 °C.

### 2.4. RNA Processing

Milk was skimmed by centrifugation for 20 min at 4 °C at 800 rpm and the lipid fraction was used for RNA extraction [21,26]. RNA was purified, sequenced, and analyzed as previously described [26,27]. The 30 miRNA features with the most robust expression (present in raw counts >10 in all 453 samples) were quantile normalized and mean-center scaled.

### 2.5. Cell Culture and let-7g-5p Transfection

Mouse mammary epithelial (HC11) cells were gifted by Dr. Jeffrey Rosen (Department of Molecular and Cellular Biology, Baylor College of Medicine, Houston, TX, USA) and used with permission of Dr. Bernd Groner (Institute for Biomedical Research, Frankfurt, Germany). Cells were maintained in RPMI 1640 growth medium supplemented with 10% fetal bovine serum, 5 μg/mL insulin (Sigma-Aldrich, Burlington, MA, USA), 10 ng/mL EGF (EMD Millipore, Burlington, MA, USA) and 1% Penicillin-Streptomycin (Sigma-Aldrich, Burlington, MA, USA). Cells were plated in antibiotic-free growth medium in 6-well plates (2.7 × 10^5^ cells/well) and cultured for 2 d. Cells were transfected for 5 h at 50% confluency with 10 nM or 30 nM of hsa-let-7g-mirVana miRNA mimic in antibiotic-free Opti-MEM reduced serum medium (ThermoFisher, Waltham, MA, USA) using Lipofectamine RNAiMAX (ThermoFisher, Waltham, MA, USA). Opti-MEM was replaced with antibiotic-free growth medium and total protein was extracted from transfected cells 25 h later for immunoblotting.

### 2.6. Immunoblotting

Cells were washed with ice-cold PBS, scraped into a RIPA lysis buffer containing protease/phosphatase inhibitors and vortexed every 5 min for 30 min until well-solubilized. The cell extract was centrifuged for 20 min at 12,000× *g* (4 °C) to pellet cellular debris and the supernatant was collected. Protein concentration was determined using the Pierce BCA protein assay kit (ThermoFisher, Waltham, MA, USA). Protein (20 μg) was solubilized in Laemmli sample buffer containing dithiothreitol (DTT; 100 mM) at 95 °C for 5 min. Proteins were separated by SDS-PAGE for 1 h (200 V) and transferred to nitrocellulose for 1 h (100 V). Immunoblotting was performed with prolactin receptor (PRLR) polyclonal antibody (1:5000; ThermoFisher, Waltham, MA, USA) and anti-rabbit horseradish peroxidase (HRP)-conjugated IgG antibody (1:10,000) or anti-IGF2BP1/IMP1 antibody (1:1000; generous gift from Dr. S. Andres, Oregon Health and Science University, Portland, OR, USA) and donkey anti-goat HRP-conjugated antibody (1:5000). The membrane was stripped with Restore Plus Western Blot Stripping Buffer (ThermoFisher, Waltham, MA, USA) and re-probed using monoclonal anti-beta actin antibody (1:8000) and anti-mouse HRP-conjugated IgG antibody (1:20,000). Proteins were visualized by chemiluminescence using SuperSignal West Femto Maximum Sensitivity Substrate (ThermoFisher, Waltham, MA, USA). Relative band intensity was quantified using ImageJ software.

### 2.7. Statistical Methods

Medical and demographic traits were compared between LMS and AMS groups using a student’s *t*-test or chi-square test, as appropriate. Levels of miRNAs in the initial milk sample (at 1 wk) were compared between LMS and AMS groups using a Wilcoxon Rank Sum test. *p*-values were adjusted for multiple testing using the false detection rate (FDR) method, and values less than 0.05 were considered significant. Candidate miRNAs identified on Wilcoxon Rank Sum testing underwent the following secondary analyses: (1) Relationships between milk miRNA levels and milk production (oz/day) were assessed with Spearman Rank Correlation testing; (2) longitudinal changes in milk levels of miRNA candidates were assessed across 1, 4, and 16 wks post-delivery using a linear mixed model fit by restricted maximum likelihood (with each miRNA as the dependent variable, participant ID as the clustering variable, and maternal characteristics as covariates). Interactions between LMS/AMS group and time (wks post-delivery) were assessed with fixed effects omnibus tests; (3) the effects of modifiable maternal characteristics (nutrition, BMI, tobacco use) on milk levels of candidate miRNAs were assessed with a mixed model (with miRNA level was the dependent variable, participant ID as the clustering variable, and maternal characteristics as covariates); (4) the ability of candidate miRNA levels to differentiate participants at risk for LMS was assessed relative to maternal factors with a hierarchical logistic regression. LMS/AMS group served as the dependent variable, and candidate miRNAs without collinearity were used in a feed-forward model building approach. Sensitivity, specificity, and area under a receiver operator characteristic curve were reported; (5) physiologic functions of the candidate miRNAs were explored in DIANA miRPath v3 [28], through identification of putative mRNA targets (Tarbase algorithm). Enrichment of Kyoto Encyclopedia Genes and Genomes (KEGG) pathways was compared to that expected by chance using a Fisher’s exact test with FDR correction.

For cell experiments, data represent mean ± SD. Statistical analysis was carried out using GraphPad Prism software (Version 9.0; GraphPad Prism Software, Inc., San Diego, CA, USA). Differences between means were determined by students t-test and were considered statistically significant at *p*-value < 0.05.

## 3. Results

### 3.1. Participants

Participating mothers were, on average 30 (±4) years of age, with a mean BMI of 27 (±6) kg/m^2^ (Table 1). The majority were white (135/170; 79%), married (134/170; 78%), multi-parous (120/170; 70%), had never used tobacco (144/170; 84%), and had obtained a college or post-graduate degree (121/170; 71%). Few experienced gestational diabetes (20/170, 11%). Approximately half had previously breastfed more than four months (90/170; 52%). Compared to mothers with AMS, mothers with LMS were older (d = 0.39, *p* = 0.023), were more likely to have a post-graduate degree (*X*^2^ = 12.1, *p* = 0.017), and were less likely to have previously breastfed (*X*^2^ = 11.9, *p* = 0.018). There were no differences in other medical or demographic factors.

### 3.2. Milk Production and Infant Weight Trajectory

Infants of mothers with LMS had a similar mean weight-for-length z-score at birth (−0.76 ± 1.0) as infants of mothers with AMS (−0.71 ± 1.1; d = 0.03, *p* = 0.41; Table 2). On average, mothers with LMS first reported difficulties with milk supply 3 (±3) months after delivery. However, 4 wks after delivery, infants of mothers with LMS displayed a lower mean weight-for-length z-score (0.05 ± 1.2) than infants of mothers with AMS (0.50 ± 1.1; d = 0.38, *p* = 0.013). At 4 wks, mothers with LMS reported lower volumes of daily milk production (20.6 ± 11.8 oz/day) than mothers with AMS (28.1 ± 15.6 oz/day; d = 0.50, *p* = 0.003), and they were more likely to have introduced formula (28/47, 59%) than mothers with AMS (27/127, 21%; *X*^2^ = 27.8, *p* = 2.1 × 10^−7^). Mothers with LMS continued to report lower volumes of daily milk production at 16 weeks (20.1 ± 14.1 oz/day) than mothers with AMS (33.3 ± 24.0 oz/day; d = 0.60, *p* = 0.0007). Mothers with LMS were also more likely to have ceased breast feeding completely at 24 wks (18/47, 38.2%), compared to mothers with AMS (22/127, 17%; *X*^2^ = 11.4, *p* = 0.0003).

### 3.3. Milk miRNA Profiles

**Figure 2:**
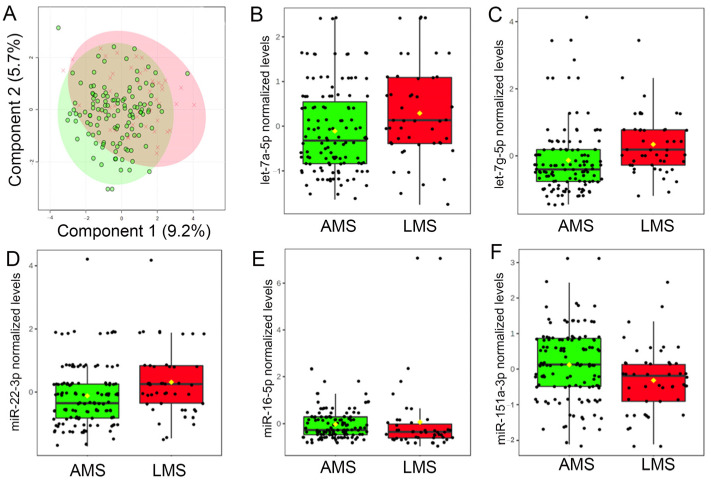
Levels of miRNAs in breast milk differ among women with low milk supply. (**A**) Results of a two-dimensional partial least squares discriminant analysis (PLSDA) employing levels of 30 miRNAs with the most robust concentrations in maternal breast milk in the first week after delivery. Note that total milk miRNA profiles do not differ between mothers with adequate milk supply (AMS; green) and mothers with low milk supply (LMS; red). However, milk levels of five miRNAs did differ in the milk of mothers with LMS. In the first week after delivery, levels of (**B**) let-7a-5p (V = 2194, *p* = 0.015, adj *p* = 0.090), (**C**) let-7g-5p (V = 1765, *p* = 8.5 × 10^−5^, adj *p* = 0.0025), and (**D**) miR-22-3p (V = 2135, *p* = 0.0080, adj *p* = 0.080) were higher in the milk of mothers with LMS, whereas levels of (**E**) miR-16-5p (V = 0.3595, *p* = 0.013 adj *p* = 0.090) and (**F**) miR-151a-3p (V = 3670, *p* = 0.0063, adj *p* = 0.080) were lower.

There was no discernible difference between the overall milk miRNA profiles the first week after delivery (Figure 2A). However, 5 of the 30 most robustly expressed milk miRNAs (i.e., >10 counts in all 453 samples), displayed nominal differences (raw *p* ≤ 0.01) between groups (Figure 2B–F). Maternal estimates of daily milk production were associated with levels of three miRNAs: miR-16-5p (R = −0.14, *p* = 0.0088, adj *p* = 0.044), miR-22-3p (R = 0.13, *p* = 0.011, adj *p* = 0.044), and let-7g-5p (R = 0.12, *p* = 0.023, adj *p* = 0.046). Only levels of let-7g-5p survived multiple testing correction (V = 1765, adj *p* = 0.0025), indicating higher levels in the milk of mothers with LMS.

### 3.4. Longitudinal Changes in LMS-Related miRNAs

**Figure 3:**
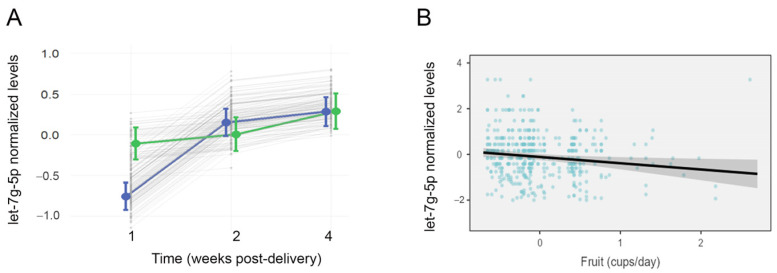
Milk levels of let-7g-5p differ over time among mothers with low milk supply and are related to maternal fruit intake. The effects plot displays normalized concentrations of let-7g-5p in 453 samples from 170 lactating mothers across three time points: 1 wk, 4 wks, and 16 wks after delivery (**A**). Mean concentrations are shown for mothers with low milk supply (LMS, green), and mothers with adequate milk supply (AMS, blue). There was a significant interaction effect (*p* < 0.001) between LMS/AMS group and time (F = 7.5), with let-7g-5p levels increasing between 1 wk and 4 wks post-delivery in the AMS group only. (**B**) A second mixed effects model also revealed a significant effect of maternal fruit consumption on milk levels of let-7g-5p (F = 5.99, *p* = 0.015). Higher levels of let-7g-5p were associated with lower fruit consumption, as reported longitudinally on the Dietary Screener Questionnaire.

Linear mixed models were used to assess candidate miRNAs for changes in abundance over the course of lactation, while controlling for maternal age, education, and breastfeeding experience. Milk levels of let-7g-5p increased over the course of lactation (F = 21.4, *p* < 0.001), and there was a significant interaction between lactation period and group (F = 7.5, *p* < 0.001; Figure 3A). Levels of let-7g-5p generally increased in the AMS group over time but remained stable in mothers with LMS. Levels of let-7a-5p (F = 15.7, *p* < 0.001), miR-22-3p (F = 71.2, *p* < 0.001), and miR-16-5p (F = 62.7, *p* < 0.001) also changed over the course of lactation but did not display an interaction between lactation stage and milk supply.

### 3.5. Effect of Modifiable Maternal Characteristics on LMS-Related miRNAs

Mixed models were used to assess the effect of nutrition, BMI, and tobacco use on candidate miRNAs. Nutrition displayed an association with milk miRNA levels over the course of lactation. Lower milk levels of let-7g-5p were associated with higher maternal fruit consumption (F = 5.99, *p* = 0.015, Figure 3B). There was no interaction between fruit consumption and milk supply (F = 0.22, *p* = 0.63). Higher milk levels of miR-22-3p were associated with lower consumption of calcium (F = 7.31, *p* = 0.007) and dairy (F = 6.48, *p* = 0.011).

### 3.6. Predicting Low Milk Supply Status

Hierarchical logistic regression was used to assess the ability of milk miRNAs to predict low milk supply, relative to medical and demographic traits. The three maternal characteristics that differed between mothers with LMS and mothers with AMS (i.e., age, education, breastfeeding experience) accounted for 15.5% of the variance between groups, (*X*^2^ = 31.0, *p* < 0.001), and accurately identified 35/47 mothers with AMS (74% sensitivity) and 80/123 mothers with AMS (65% specificity; AUC = 0.763). Addition of three miRNAs that lacked co-linearity (i.e., miR-22-3p, let-7a-5p, and let-7g-5p) accounted for an additional 8.1% of variance between groups (*X*^2^ = 47.3, *p* < 0.001), and significantly improved the model (*X*^2^ = 16.2, *p* = 0.001). The combined model displayed an AUC of 0.816.

### 3.7. Pathway Analysis

The messenger RNA targets of the five miRNAs of interest (miR-22-3p, miR-16-5p, let-7a-5p, let-7g-5p, miR-151a-3p) were interrogated in DIANA miRPATH using the Tarbase algorithm. The five miRNAs targeted 13 physiologic pathways with greater frequency than would be expected by chance alone, including cell cycle, fatty acid biosynthesis, adherens junctions, Hippo signaling, TGFβ signaling, and ECM-receptor interaction (Table 3).

**Figure 4:**
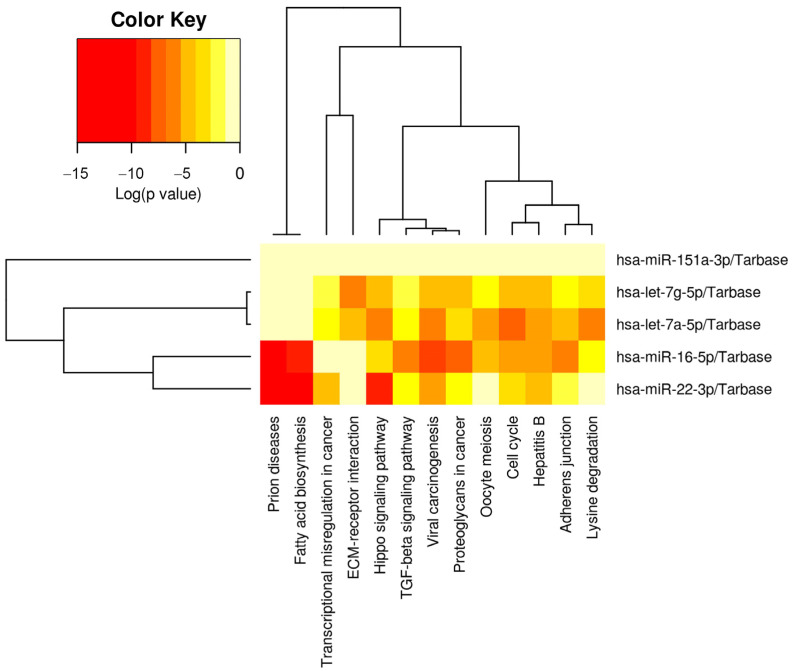
Physiologic pathways targeted by the five milk miRNAs implicated in low milk supply. The heatmap displays the relative level of messenger RNA targets for 13 Kyoto Encyclopedia of Genes and Genomes (KEGG) pathways, where red represents the highest number of targets. These 13 pathways all displayed a significantly greater number of targets than would be expected by chance alone on Fisher Exact Testing. Note that pathways and miRNAs have been clustered using the Ward method, and the dendrograms display relatedness of each pathway or miRNA.

Hierarchical clustering showed that let-7a-5p and let-7g-5p had the most closely related physiologic targets, and miR-22-3p and miR-16-5p displayed the second highest physiologic relatedness (Figure 4).

Due to the interaction between let-7g-5p, lactation stage, and milk supply, we further queried molecular pathways affected by this miRNA. Key KEGG pathways predicted to be downregulated by let-7g-5p (https://genome.jp, accessed on 2 November, 2022) include Metabolic, PI3K-Akt signaling, MAPK signaling, and JAK-STAT signaling pathways (Table 4), and include numerous genes associated with lactation traits such as those involved in hormone signaling (*PRLR*, *INSR)*, extracellular matrix (*COL1A1-2, COL3A1, COL4A1-3* and *A6, COL5A2, COL14A1, COL27A1*), zinc transport (*SLC30A4*), H^+^ transport (*ATP6V1G1, ATP6V1C1*), and calcium transport (*ATP2A2, ATP2B4*). Moreover, the top mRNAs identified included several novel lactogenic targets involved in mRNA binding (*IGF2BP1-3*), chromatin binding (*HMGA2*), and actin assembly *(STARD13)* (Supplementary Materials).

### 3.8. Confirmation of PRLR and IGF2BP1 Regulation in Mammary Cells

**Figure 5:**
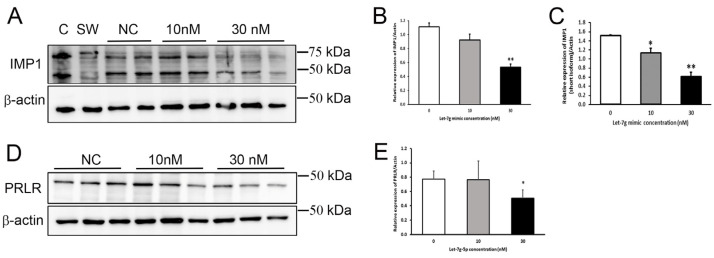
Let-7g-5p reduced IGF2BP1/IMP1 and prolactin receptor (PRLR) expression in mammary epithelial (HC11) cells. (**A**) Representative immunoblot of IGF2BP1/IMP1 in protein lysates (20 μg protein/well) from HC11 cells transfected with 10 nM or 30 nM of let-7g-5p mimic compared with non-transfected (NC) cells. Caco-2 (**C**) and SW480 (SW) protein lysates were used as positive controls. Membranes were striped and re-probed for β-actin. (**B**) Relative expression of the long form of IGF2BP1/IMP1 (~70 kDa) after normalization to β-actin. Results represent the mean ratio ± SD (*n* = 2–3 samples/group). ** *p* < 0.01 (**C**) Relative expression of the short form of IGF2BP1/IMP1 (~42 kDa) after normalization to β-actin. Results represent the mean ratio ± SD (*n* = 2–3 samples/group). * *p* < 0.05, ** *p* < 0.01 (**D**) Representative immunoblot of PRLR in protein lysates (20 μg protein/well) from HC11 cells transfected with 10 nM or 30 nM of let-7g-5p mimic compared with non-transfected (NC€ells. (**E**) Relative PRLR protein expression of after normalization to β-actin. Results represent the mean ratio ± SD (*n* = 3 samples/group) from two separate experiments. * *p* < 0.05.

To directly confirm the effect of elevated levels of let-7g-5p in MECs, cells were transiently transfected with a let-7g-5p mimic. Protein expression of two predicted mRNA targets, PRLR and IGF2BP1/IMP1, was measured 30 h later by immunoblot (Table S1). PRLR was selected for confirmation of its regulation by let-7g-5p in MECs because of its well-known importance in lactation, and IGF2BP1/IMP1 was selected to assess effects on a novel molecular target. Both the long and short forms of IGF2BP1/IMP1 were identified in HC11 cells [29], and both isoforms were significantly lower (*p* < 0.01) in cells transfected with 30 nM hsa-let-7g mimic compared to non-transfected cells (Figure 5A–C). In addition, PRLR protein expression was significantly lower in cells transfected with 30 nM hsa-let-7g mimic (*p* < 0.05) compared to non-transfected cells (Figure 5D,E). These results confirm that expression of PRLR and IGF2BP1/IMP1 are negatively regulated by let-7g-5p in MECs and provide evidence of two discrete molecular mechanisms that may underlie effects of let-7g-5p on lactation and milk supply.

## 4. Discussion

Here, we present a novel approach for identifying biological factors that underpin low milk supply in breastfeeding women. We established that the profile of milk-derived miRNAs reflects lactation performance in humans, and for the first time identified miRNAs associated with low milk supply. Importantly, we determined that let-7g-5p may serve as a potential regulon of breast function and predicted key genes involved in metabolism, hormone signaling, ion transport, mRNA binding, and tissue remodeling in the lactating mammary gland, supporting previous studies that have posited a role for let-7g-5p in breast function [30]. Importantly, bioinformatic prediction of PRLR and IGF2BP1/IMP1 as let-7g-5p targets was confirmed in MECS. We detected a significant interaction between let-7g-5p, milk volume, and lactation stage, suggesting that measurement of let-7g-5p levels in milk during early lactation may be useful in predicting risk for low milk supply. Interestingly, our data suggest the let-7g-5p regulon may be modifiable as there was a negative relationship between let-7g-5p levels and fruit intake in our population of breastfeeding women.

Milk is one of the richest sources of miRNAs in humans and contains ~1400 mature miRNAs [17] and the profile of milk miRNAs has been proposed to reflect mammary gland function [17,18,19,20,21]. We found maternal estimates of daily milk production were indeed associated with levels of miR-16-5p, miR-22-3p, and let-7g-5p; however, only levels of let-7g-5p survived multiple testing correction. The *let-7* family of miRNAs is one of the earliest discovered miRNA clusters and is conserved across species [31]. It is comprised of ten miRNAs (*let-7a, let-7b, let-7c, let-7d, let-7e, let-7f, let-7g, let-7i, miR-98,* and *miR-202*) and plays key roles in suppressing proliferation and differentiation [32] and is a key regulator of glucose metabolism [33]. Let-7g-5p levels were enriched in the milk of women with low milk supply, consistent with a putative role for let-7g-5p suppression in motivating proliferation, suppressing mammary gland differentiation [30] and affecting mammary gland metabolism. Importantly, let-7g-5p is conserved between human and mouse [31], suggesting the opportunity to use preclinical mouse models to understand mechanistic implications of let-7g-5p on MEC proliferation, differentiation, and milk production and secretion.

A critical gap in knowledge is how let-7g-5p is regulated in mammary epitheial cells. Let-7g is found on chromosome 3 in humans [31] and is post-transcriptionally repressed through binding of the RNA binding protein Lin28 to its terminal loop, thereby either inhibiting binding of Dicer and Drosha or re-routing pre-let-7g for degradation [34]. Thus, factors that affect Lin28 regulation would have major impacts on mammary gland function. One potential factor that regulates the Lin28/let-7 axis is inflammation [35]. Interestingly, inflammation is known to compromise lactation and milk supply [36,37], thus therapeutic strategies to reduce inflammation may be key to maximizing milk supply. Additionally, let-7g expression is repressed by DNA methylation [38], suggesting intake of methyl donors such as folate [39] and B_12_ may play a role. Several studies suggest estrogen may regulate let-7g-5p; however, results are inconsistent [40,41]. Positive regulation by estrogen would be consistent with suppression of let-7g-5p levels upon estrogen withdrawal at partition and the subsequent activation of lactogenesis II. Given the importance of robust activation lactogenesis II in the maintenance of copious milk production going forward, identification of factors that regulate let-7g-5p is critical to our understanding of this regulon and its intriguing role in lactation [42].

Numerous mRNA targets of let-7g-5p associated with lactation traits include hormone signaling (*PRLR*, *INSR*) [43,44,45], extracellular matrix (*COL1A1-2, COL3A1, COL4A1-3* and *A6, COL5A2, COL14A1, COL27A1*) [46], zinc transport (*SLC30A4*) [47], H^+^ transport (*ATP6V1G1, ATP6V1C1*) [48], and calcium transport (*ATP2A2, ATP2B4*) [49], further implicating it as a regulatory hub for maintaining milk supply. Importantly, our study confirmed that PRLR expression was indeed regulated by let-7g-5p in MECs, providing direct evidence that let-7g-5p is a critical regulator of lactation. Three examples of novel targets of let-7g-5p that may have potential lactogenic implications include steriodogenic acute regulatory protein-related lipid transfer domain-containing protein 13 (STARD13), high mobility group AT-hook 2 (HMGA2), and insulin-like growth factor 2 messenger RNA-binding protein (IGF2BP1/IMP1). STARD13 serves as a Rho-GTPase activating protein that selectively regulates RhoA and cdc42 to inhibit actin assembly. STARD13 attenuation leaves RhoA constitutively active which inhibits Rac and thus inhibits motility [50]. To our knowledge, a role for STARD13 in mammary gland development or lactation has yet to be explored; however, this finding may have important implications for breast cancer detection as STARD13 is a tumor suppressor and Rac1 plays a major role in cancer cell motility [50]. HMGA2 is a group of small chromatin-associated proteins that act as an architectural transcription factor that directly binds to DNA and modulates the transcription of target genes; however, a putative role for HMGA2 in the breast requires exploration. IGF2BP1/IMP1 is a highly conserved RNA-binding protein that regulates RNA processing at several levels, including localization, translation, and stability. Key targets of IGF2BP1/IMP1 with potential consequences on lactation include β-actin, STAT3, c-myc, glutathione peroxidases, and several mitochondrial proteins [51]. Two IGF2BP1/IMP1 isoforms have been identified in MECs [29], a long isoform (~70 kDa) and a short isoform (~40 kDa) resulting from an *N*-terminal truncation with currently unknown function. Herein, we confirmed that let-7g-5p downregulated both isoforms, suggesting key molecular functions such as morphogenesis, oxidative stress, and ATP production are targets of the let-7g-5p regulon, which implicates IGF2BP1/IMPs as a novel molecular target for low milk supply.

While our genome-wide approach identified critical milk-derived miRNAs and predicted novel genes and molecular pathways for further exploration, an exciting finding from this study was that high milk levels of let-7g-5p during early lactation may be useful as a bioreporter of low milk supply and risk for early breastfeeding cessation. This observation warrants further study as >40% of women cite concerns over low milk supply as a primary reason for not meeting their breastfeeding goals [1] and early identification could inform interventions to improve breastfeeding success. One such intervention might include dietary modification, as intriguing findings suggest levels of let-7g-5p may be modifiable. For example, ursolic acid (UA) is a natural triterpene found in various fruits and vegetables that suppresses let-7g-5p [52] and there is a growing interest in UA because of its beneficial effects, which include pro- and anti-inflammatory, antioxidant, anti-apoptotic, and anti-carcinogenic effects [53]. Additionally, quercetin is a polyphenol ubiquitously present in certain fruits (e.g., apples and grapes) and vegetables (e.g., onions, kale, broccoli, lettuce, and tomatoes) that also has pro- and anti-inflammatory and antioxidant capacity, and let-7g-5p levels are positively associated with a quercetin-rich diet [54]. Further studies are required to reproduce our findings and determine how dietary polyphenols and secondary metabolites affect the let-7g-5p regulon in the mammary gland, which may eventually offer a therapeutic option with an evidence-based rationale for women with low milk supply.

There are several strengths of the present study: (1) a large sample size with longitudinal collections; (2) uniformity insample collection and processing; (3) high throughput sequencing; and (4) the use of mixed effects models to assess relative impacts of maternal characteristics. However, there are several limitations. The scatter in our data suggest that trends may be driven more heavily by samples at the fringe ends of the dataset, and additional studies are required to confirm our findings. The current cohort was mostly white and included only mothers delivering at term, which may limit generalizability of the findings. In addition, given our finding that let-7g-5p may be a potential regulator, further studies should include potential modifiers such as caloric intake, physical activity, quality of life, and comprehensive dietary analysis to better understand the role and regulation of let-7g-5p during lactation. Finally, our results (which include both exosomal and non-exosomal miRNA) may differ from studies focused solely on exosomal miRNAs [55] and while prior studies have demonstrated minimal miRNA differences across milk fractions [56,57], results from miRNAs in skim versus cellular fractions may differ.

## 5. Conclusions

In conclusion, the results of this study advance our collective understanding of the biological contributors to low milk supply by identifying miRNAs, novel molecular pathways, and new gene targets associated with poor milk production. Predicted transcripts highlight both classical and novel lactogenic targets, therefore future studies should interrogate consequences of these miRNAs on mammary gland function. Importantly, this study is the first to identify an interaction between milk levels of miRNAs, low milk supply, and early cessation of breastfeeding, and suggests that measuring milk levels of let-7g-5p during the first weeks after birth may be a useful tool in predicting risk for low milk supply and identifying women who need targeted lactation support.

## Figures and Tables

**Table 1:** Participant characteristics.

	All (*n* = 170)	AMS (*n* = 123)	LMS (*n* = 47)
Age in years, mean (SD)	30 (4.6)	29.5 (4.5)	31.3 (4.7) *
Asian, *n* (%)	8 (4.7)	6 (4.9)	2 (4.3)
Bi-racial, *n* (%)	6 (3.5)	4 (3.3)	2 (4.3)
African American, *n* (%)	12 (7.1)	8 (6.5)	4 (8.5)
Other—not specified, *n* (%)	3 (1.8)	3 (2.4)	0 (0.0)
Other—specified, *n* (%)	6 (3.5)	4 (3.3)	2 (4.3)
White, *n* (%)	135 (79.4)	98 (79.7)	37 (78.7)
Parity, # (SD)	2 (1)	2 (1)	2 (1)
Pre-pregnancy BMI, kg/m^2^, mean (SD)	27.8 (6.7)	27.2 (6.1)	29.2 (8.0)
Gestational diabetes, *n* (%)	20 (11.8)	13 (10.6)	7 (14.9)
Prior tobacco use, *n* (%)	26 (15.3)	17 (13.8)	9 (19.1)
Some HS, *n* (%)	2 (1.2)	2 (1.6)	0 (0.0)
Completed HS, *n* (%)	22 (12.9)	19 (15.4)	3 (6.4) *
Some college, *n* (%)	25 (14.7)	16 (13.0)	9 (19.1)
Completed college, *n* (%)	69 (40.6)	56 (45.5)	13 (27.7)
Post-graduate degree, *n* (%)	52 (30.6)	30 (24.4)	22 (46.8) *
Single, *n* (%)	11 (6.5)	9 (7.3)	2 (4.3)
Divorced, *n* (%)	2 (1.2)	1 (0.8)	1 (2.1)
Co-habitating, *n* (%)	23 (13.5)	16 (13.0)	7 (14.9)
Married, *n* (%)	134 (78.8)	97 (78.9)	37 (78.7)
Never, *n* (%)	67 (39.4)	42 (34.1)	25 (53.2) *
<1 month, *n* (%)	4 (2.4)	1 (0.8)	3 (6.4)
1–2 months, *n* (%)	4 (2.4)	4 (3.3)	0 (0.0)
2–4 months, *n* (%)	5 (2.9)	4 (3.3)	1 (2.1)
>4 months, *n* (%)	90 (52.9)	72 (58.5)	18 (38.3) *
Gestational age, weeks (SD)	38.9 (1.0)	38.9 (1.0)	39.0 (1.0)
Infant Sex, female, *n* (%)	99 (58.2)	74 (60.1)	25 (53.1)
Birth weight, grams (SD)	3364 (440)	3377 (440)	3329 (443)

* Denotes *p* < 0.05 on Student’s *t*-test or chi-square test. Abbreviations: Body mass index (BMI); High school (HS).

**Table 2:** Infant weight trajectory and feeding characteristics.

	All (*n* = 170)	AMS (*n* = 123)	LMS (*n* = 47)
Formula intro by 4 weeks, *n* (%)	55 (32.3)	27 (21.9)	28 (59.5) *
Breastfeeding at 6 months, *n* (%)	134 (78.8)	105 (85.4)	29 (61.7) *
Milk production at 4 weeks, oz/day (SD)	26.0 (15.0)	28.1 (15.6)	20.6 (11.8) *
Milk production at 16 weeks, oz/day (SD)	29.6 (22.5)	33.3 (24.0)	20.1 (14.1) *
Infant WfL Z-score at birth	−0.72 (1.1)	−0.71 (1.1)	−0.76 (1.0)
Infant WfL Z-score at 4 weeks	0.38 (1.2)	0.50 (1.1)	0.05 (1.2) *
∆ WfL Z-score from birth to 4 weeks	1.1 (1.4)	1.2 (1.4)	0.8 (1.3) *

* *p* < 0.05 on Student’s *t*-test or chi-square test. Abbreviations: Introduction (intro), Weight for length (WfL).

**Table 3:** Physiologic pathways targeted by the five candidate miRNAs.

KEGG Pathway	*p*-Value	Genes (#)	miRNAs (#)
Prion diseases	6.88 × 10^−17^	13	2
Cell cycle	1.11 × 10^−16^	63	3
Hepatitis B	3.77 × 10^−15^	72	3
Proteoglycans in cancer	4.77 × 10^−14^	85	2
Fatty acid biosynthesis	1.63 × 10^−13^	4	2
Adherens junction	1.29 × 10^−11^	46	2
Hippo signaling pathway	1.42 × 10^−10^	57	3
TGF-beta signaling pathway	1.32 × 10^−09^	31	1
Lysine degradation	1.77 × 10^−09^	15	1
Oocyte meiosis	6.19 × 10^−09^	35	1
Viral carcinogenesis	9.29 × 10^−9^	102	4
ECM-receptor interaction	8.02 × 10^−08^	16	2
Transcriptional misregulation	2.10 × 10^−05^	38	1
in cancer			

*p*-values represent multiple correction adjusted *p*-values on Fisher’s exact *t*-tests. The number of genes targeted by each of the five miRNAs was determined using the Tarbase algorithm in DIANA miRPath software. The top 13 pathways (adj. *p* < 1.0 × 10^−5^) are shown.

**Table 4:** Top twenty physiologic pathways targeted by let-7g-5p.

KEGG Pathway	Genes (#)
Metabolic pathways	72
Pathways in cancer	39
PI3K-Akt signaling pathway	33
Human Papillomavirus infection	30
Herpes simplex virus infection	29
Pathways of neurodegeneration	28
Proteoglycans in cancer	23
MicroRNAs in cancer	23
Focal adhesion	21
Axon guidance	21
Human T-cell leukemia virus 1 infection	20
Cytokine-cytokine receptor interaction	20
Transcriptional misregulation in cancer	19
FOXO signaling pathway	19
Ras signaling pathway	19
Calcium signaling pathway	18
mTOR signaling pathway	18
JAK-STAT signaling pathway	18
Lipid and atherosclerosis	18
AGE-RAGE signaling pathway	18

## Data Availability

The RNA sequencing data presented in the study are deposited in the Gene Expression Omnibus repository, accession number GSE192543. GEO repository link: https://www.ncbi.nlm.nih.gov/geo/query/acc.cgi?acc=GSE192543; 1 January, 2022.

## Supplementary Materials

The following supporting information can be downloaded at: https://www.mdpi.com/article/10.3390/nu15030567/s1, Table S1: predicted mRNA targets.
